# In Situ Water Quality Monitoring for the Assessment of Algae and Harmful Substances in Water Bodies with Consideration of Uncertainties

**DOI:** 10.3390/s25227055

**Published:** 2025-11-19

**Authors:** Stefanie Penzel, Thomas Mayer, Helko Borsdorf, Mathias Rudolph, Olfa Kanoun

**Affiliations:** 1 Helmholtz Centre for Environmental Research—UFZ, Permoserstraße 15, 04318 Leipzig, Germany; 2 Leipzig University of Applied Sciences, Karl-Liebknecht-Straße 132, 04277 Leipzig, Germany; 3 Chemnitz University of Technology, Reichenhainer Straße 9, 09126 Chemnitz, Germany

**Keywords:** water quality monitoring, in situ, harmful algae, UV/Vis and fluorescence spectroscopy, uncertainties

## Abstract

Harmful algal blooms, particularly those caused by cyanobacteria (blue-green algae) and green algae, pose an increasing risk to aquatic ecosystems and public health. This risk is intensified by climate change and nutrient pollution. This study presents a methodology for in situ monitoring and assessment of algal contamination in surface waters, combining UV/Vis and fluorescence spectroscopy with a fuzzy pattern classifier for consideration of uncertainties. The system incorporates detailed data pre-processing to minimise measurement uncertainty and uses full-spectrum feature extraction to enhance classification accuracy. To assess the methodology under both controlled and real-world conditions, a mobile submersible probe was tested alongside a laboratory setup. The results demonstrate a high degree of agreement between the two systems, showing particular sensitivity to biological signals, such as the presence of algae. The assessment method successfully identified cyanobacterial and green algal contamination, and its predictions aligned with external observations, such as official warnings and environmental changes. By explicitly accounting for measurement uncertainty and employing a comprehensive spectral analysis approach, the system offers robust and adaptable monitoring capabilities. These findings highlight the potential for scalable, field-deployable solutions for the early detection of harmful algal blooms.

## 1. Introduction

The increasing amount of pollutants in surface water, ranging from industrial effluents and agricultural runoff to urban and domestic waste, poses a serious threat to the environment and public health. These stressors degrade aquatic ecosystems and compromise access to clean water, which is a critical resource for human well-being and economic resilience. Climate-related factors, such as prolonged droughts, rising temperatures, and changes in hydrological patterns, further exacerbate these issues by affecting water quality and encouraging the growth of harmful algal blooms [1,2].

As these challenges become increasingly complex and pressing, there is an urgent need for reliable and rapid on-site monitoring techniques that can detect critical water quality parameters. However, accurately characterizing contaminated water systems remains a significant scientific and technical challenge, particularly in dynamic environmental conditions. Optical methods such as ultraviolet-visible (UV/Vis) and fluorescence spectroscopy have therefore emerged as powerful tools for rapidly and non-invasively analyzing water quality. These spectroscopic techniques enable a non-invasive and rapid assessment of various water quality parameters and are particularly valuable for both qualitative and quantitative evaluations. Their ability to detect a wide range of organic and inorganic substances, dissolved solids, and even indicators for microbial contamination makes them essential tools in environmental monitoring. UV/Vis and fluorescence spectroscopy are characterized by their high sensitivity and relatively low operational costs, making them suitable for continuous and large-scale monitoring scenarios [3,4,5]. In particular, fluorescence spectroscopy offers enhanced selectivity for specific organic compounds, while UV/Vis spectroscopy is frequently used to derive surrogate parameters based on characteristic absorption patterns [4]. These technologies are widely used in field-based applications, including the deployment of submersible sensors for in situ analysis. This facilitates real-time monitoring and decision-making, especially in the context of wastewater treatment, surface water observation, and ecological studies [3,6]. Moreover, recent research has demonstrated the potential of combining spectroscopic data with data-driven approaches such as fuzzy logic and machine learning. These methods allow for improved interpretation of complex spectral datasets and have been successfully applied in scenarios such as algal detection and classification of water contaminants [7,8]. Overall, UV/Vis and fluorescence spectroscopy represent robust and cost-efficient techniques for water quality monitoring. Their integration with intelligent evaluation strategies is expected to further expand their capabilities in automated and adaptive environmental monitoring systems [5].

While UV/Vis and fluorescence spectroscopy offer significant advantages in terms of sensitivity, speed, and cost-effectiveness, their accuracy and reliability are strongly influenced by various sources of uncertainty. This is particularly relevant in in situ applications, where measurements are performed under real-world environmental conditions. Factors such as temperature variations, turbidity, biofouling, sensor drift, and matrix effects lead to considerable uncertainties that often do not occur in laboratory environments or are easier to control.

Table 1 provides an overview of selected scientific publications that have already addressed uncertainties in UV/Vis and fluorescence spectroscopy to varying degrees. However, it also becomes evident that these uncertainties are often not comprehensively considered. In numerous instances, they are only partially discussed or not systematically integrated into the methodological approaches. This highlights the need for a more holistic and structured treatment of uncertainty in spectroscopic water analysis, particularly when aiming for robust in situ applications.

Addressing these challenges, this study explores the potential of in situ water quality monitoring to detect and evaluate algal contamination, focusing particularly on blue-green (cyanobacteria) and green algae. Although the classification system can theoretically identify other parameters, such as turbidity and chemical pollutants, this paper exclusively evaluates its performance using water samples affected by algal blooms. The study also considers the impact of measurement uncertainties, which are a critical factor in real-world monitoring applications. The main objective is to develop and evaluate a methodology for the in situ detection and classification of algae in real environmental conditions while explicitly accounting for uncertainty in spectroscopic measurements. This approach combines UV/Vis and fluorescence spectroscopy with robust, data-driven evaluation techniques to improve the reliability of sensor-based algal detection. The results demonstrate that incorporating uncertainty-aware methods improves the interpretability of spectral data and enables more accurate algal type identification. To ensure both methodical validation and real-world applicability, measurements were initially conducted using a controlled laboratory setup. Based on the same measurement principles, a developed submersible probe is also used for the measurement of the results. Ultimately, these findings will contribute to the development of practical, scalable tools for the early warning and monitoring of harmful algal blooms, an issue that is becoming increasingly relevant in the context of growing ecological pressures and climate-related changes.

The paper is structured as follows: The Introduction outlines the background and summarizes relevant research in the field. This is followed by a detailed description of the proposed methodology for water assessment, including data acquisition, pre-processing, feature extraction, and classification with consideration of uncertainty handling. The Section 3 presents the findings of a targeted measurement campaign, which involved recording data from algae-affected waters using laboratory and in situ measurements. The subsequent discussion addresses the differences between laboratory and diving probe data collection methods and interprets the results. The article concludes with a summary of the most significant findings and their implications for future research.

## 2. Water Assessment Methodology

The methodological framework follows a structured workflow that is common in machine learning, covering the following steps: data acquisition, preprocessing, feature extraction, and classification [21,22]. Each step is customized to enable precise in situ water quality monitoring, with a focus on detecting algae and harmful substances. A key feature of the approach is the explicit incorporation of measurement uncertainties into the classification process via fuzzy pattern classification. This allows the influence of varying environmental conditions on measurement accuracy to be considered. This approach enhances robustness by effectively handling ambiguous data.

### 2.1. Data Acquisition

A custom-designed experimental setup was developed to record the absorbance and fluorescence spectra of water samples. This setup was later integrated into a mobile submersible probe for in situ measurements. It includes a miniaturized deuterium-tungsten light source (FiberLight D2, Heraeus, Hanau, Germany) for UV/Vis absorbance measurements, as well as a specially developed LED array for fluorescence and scattered light detection. The measuring cell, housed within a stainless-steel body, features two 90° collimators (PL-25-12-90SS-SLIM2-CO, Plasus, Mering, Germany) positioned opposite each other for precise optical alignment. The UV/Vis light source and spectrometer are connected to the collimators via solarization-resistant fiber optic cables (FG600AEA, Thorlabs, Newton, NJ, USA). The LED array is mounted at a 90° angle to the spectrometer to enable accurate fluorescence measurements. Light signals are detected using a Broadcom Qmini Wide UV spectrometer (San José, CA, USA), which covers a spectral range of 225–900 nm with a resolution of 1.5 nm.

**Figure 1:**
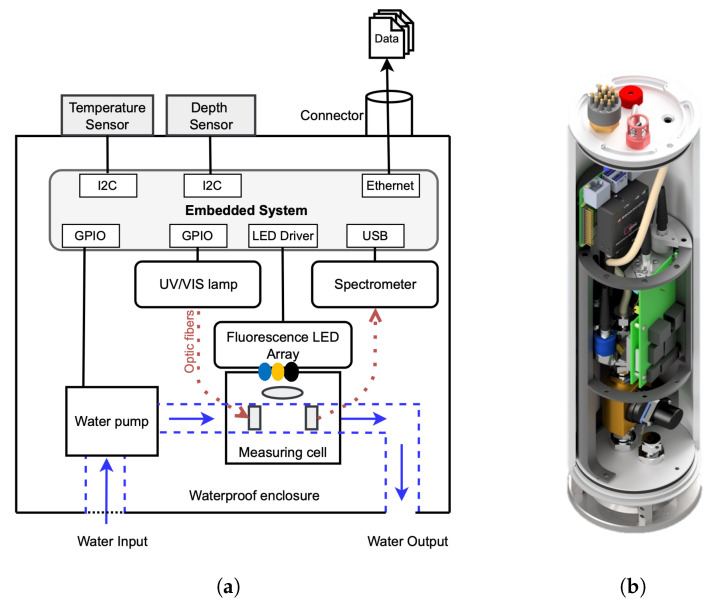
(**a**) Schematic representation of the laboratory measurement setup. (**b**) Image of the implemented submersible probe designed for in situ water quality monitoring [24].

All components are controlled by an embedded Raspberry Pi 4 Model B system, which manages the LED driver (TLC59108F, Texas Instruments, Dallas, TX, USA) via an I^2^C bus and communicates with the spectrometer via USB. The entire system is designed to minimize external interference. The probe can be submerged directly in bodies of water, where it pumps water through the measuring cell, takes a reading, and then drains the sample. Measurements can be conducted with the pump turned off to ensure stability. The configuration of the probe and the experimental setup are illustrated schematically in Figure 1, with the blue LED denoting excitation at 440 nm, the yellow LED representing excitation at 590 nm, and the black LED indicating excitation at 850 nm. It is important to note that these light-emitting diodes can also be modified. However, this study requires these excitation light sources. Further technical and software details are provided by Goblirsch et al. [23].

Reference substances for algal detection were created using dilution series prepared with spinach extract (as a source of chlorophyll) and phycocyanin (a pigment found in blue-green algae). Spinach extract was prepared using the method described in DIN EN 17899, Annex C.3, except that water was used instead of ethanol as the solvent. As the extract contains various undefined compounds, a stock solution was first prepared and subsequently diluted to achieve concentrations ranging from 0.25% to 40% of the stock solution. Spectra were recorded using UV/Vis absorbance, fluorescence (excitation at 440 nm) and scattered light (excitation at 850 nm). To ensure robust classifier development, the measurements were repeated over several days. The variations observed in the spectra, even with identical solvent compositions, were attributed to the heterogeneous nature of the spinach extract. This variability mirrors the natural variation in the composition of real-world algal samples. Furthermore, phycocyanin fluorescence spectra were recorded at an excitation wavelength of 590 nm. As cyanobacteria contain both chlorophyll and phycocyanin, the latter serves as a specific marker.

### 2.2. Data Preprocessing

The first step is to perform a temperature correction. Temperature fluctuations can occur continuously in both the laboratory and on-site in the water, which directly affects the experimental setup and measurements. To analyze the impact of temperature, the entire experimental setup was placed in a climate chamber (FTD 100), where different temperatures can be set to observe the effects of temperature changes. To compensate for the effect of temperature, a factor is determined that normalizes the spectra to a reference temperature of 21.3 °C. The influence of temperature also depends significantly on the specific light source used. Therefore, a temperature correction factor is calculated for each light source and applied automatically during data preprocessing. This correction has been verified as valid up to approximately 30 °C. Beyond this range, however, deviations may occur due to non-linear thermal effects.

In addition, the UV/Vis spectra are converted to absorbance values using Lambert’s law (see Equation (1)). At the start of each measurement, a blank spectrum of pure water is recorded. The absorbance spectrum is then obtained by subtracting this blank from the measured spectrum as part of the preprocessing step to improve the accuracy of the results. The absorbance *A* is defined as

(1) $$A=\log\left(\frac{I_{0}}{I}\right)=\varepsilon_{\lambda }cd,$$

where $I_{0}$ is the intensity of the incident light, and *I* is the transmitted light intensity. According to Lambert’s law, the transmitted intensity depends on the molar absorptivity $\varepsilon_{\lambda }$, the concentration *c*, and the path length *d*. For fluorescence measurements, the blank spectrum is also subtracted from the measured intensity *I* during preprocessing to yield the fluorescence intensity *F*:

(2) $$F=I-I_{0},$$

where $I_{0}$ is the blank intensity and *I* is the sample intensity.

To reduce noise in the spectra and improve data quality, a pre-processing filter is applied to the data. Due to the desired linear phase characteristics, a 30th-order FIR low-pass filter is used. To avoid phase shifts in the spectra, zero-phase filtering is performed using bidirectional filtering. The FIR filter is configured with a passband frequency of $f_{p}=0.1f_{s}$ and a stopband frequency of $f_{sb}=0.3f_{s}$, where $f_{s}$ is the sampling rate.

An important aspect of data pre-processing is turbidity compensation. In real-world measurements, turbidity is always present and has a significant effect on the UV/Vis spectra. The turbidity compensation method used here has been described in detail in a previous publication by Penzel et al. [24]. To account for turbidity, scattered light spectra are recorded using an excitation wavelength of 850 nm. This spectral region is highly sensitive to turbidity-induced scattered light, making it ideal for estimating turbidity levels. The intensity of the scattered light spectrum is used as a quantitative measure of turbidity (expressed in FNU) to adjust the UV/Vis spectra. Subtracting the turbidity-related contribution from the UV/Vis spectrum effectively isolates the true absorbance. The exact procedure and algorithmic implementation can be found in the referenced publication. In this study, turbidity compensation is applied to UV/Vis spectra automatically. This compensation method is valid up to turbidity levels of around 20 FTU. For higher turbidity values, the accuracy of the correction decreases due to the effects of multiple scattering.

**Figure 2:**
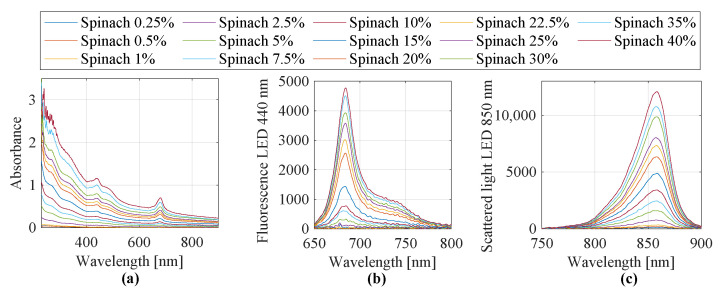
Recorded and preprocessed spinach spectra: (**a**) UV/Vis absorbance spectra, (**b**) fluorescence spectra at an excitation wavelength of 440 nm, and (**c**) scattered light spectra at an excitation wavelength of 850 nm.

Figure 2 exemplarily shows the fully preprocessed spectra of spinach extract at different dilution levels, recorded using three optical measurement methods. Displayed are (a) UV/Vis absorbance spectra, (b) fluorescence spectra with excitation at 440 nm, and (c) scattered light spectra with excitation at 850 nm. These spectra represent the final processed data used for feature extraction and subsequent classification.

### 2.3. Feature Extraction

**Figure 3:**
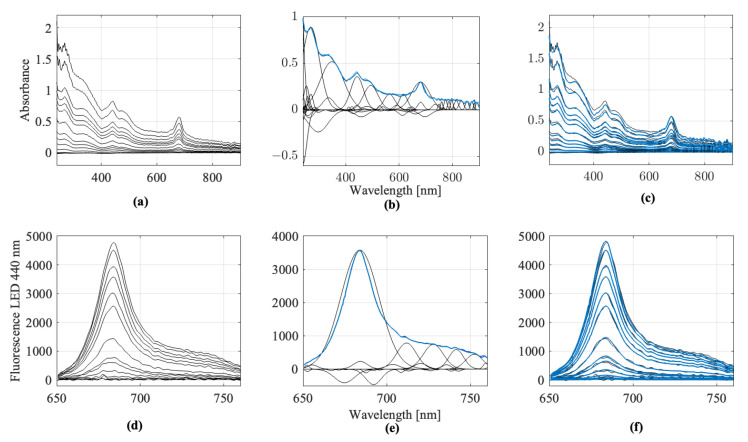
Recorded and preprocessed absorbance and fluorescence spectra of spinach samples: (**a**) Recorded and preprocessed absorbance spectra of spinach; (**b**) Generation of the reference spectrum at 25% spinach dilution; (**c**) Mathematical modelling of the recorded absorbance spectra using the reference spectrum and output of the extracted features; (**d**) Recorded and preprocessed fluorescence spectra of spinach with excitation at LED 440 nm; (**e**) Generation of the reference spectrum at 25% spinach dilution; (**f**) Mathematical modelling of the recorded fluorescence spectra using the reference spectrum and output of the extracted features.

Following preprocessing, feature extraction is performed to derive characteristic parameters from the spectra. For feature extraction, a reference spectrum is first created for each substance at a specific concentration or dilution series value. This reference spectrum $A_{\mathrm{ref},i}\left(\lambda \right)$ is composed of a sum of Gaussian functions.The reference spectrum is defined as follows:

(3) $$A_{\mathrm{ref},i}\left(\lambda \right)=k\cdot \sum_{i=1}^{N}a_{i}\cdot \exp\left(-\frac{{\left(\lambda -\lambda_{i}\right)}^{2}}{c_{i}}\right),$$

where λ is the wavelength, *N* is the number of Gaussian components, $a_{i}$ represents the amplitude (peak height), $\lambda_{i}$ the center wavelength, and $c_{i}$ the width of each Gaussian function. The scaling factor *k* accounts for the intensity variations between the spectra. These parameters are optimized iteratively by identifying local maxima in the absorbance or difference spectra and then subtracting fitted Gaussians until the residual spectrum is almost flat or contains no significant peaks. This approach enables a mathematically defined reference spectrum to be constructed for each substance (e.g., algae substance, turbidity, pure water, etc.). Consequently, each recorded spectrum can be described by its respective Gaussian-based reference spectrum. A key extracted feature is the determination coefficient $R^{2}$, which quantifies the match between the measured spectrum and the reconstructed reference. This serves as a measure of the conformity of the substance. This procedure is illustrated in Figure 3, using spinach spectra at a specific dilution level as an example. The Figure demonstrates how the fitted Gaussian functions approximate the original measurement and how the reference spectrum is constructed.

### 2.4. Fuzzy Classification

To classify the extracted spectral features while accounting for measurement uncertainties, a fuzzy pattern classification approach is applied. This method, developed by Bocklisch and colleagues [25,26], allows uncertainties (particularly those caused by varying environmental conditions) to be explicitly integrated into the classification process.

The fuzzy pattern classifier is a supervised learning method consisting of two phases: a learning phase and a working phase. During the learning phase, the extracted features are initially grouped using a conventional classification method, such as cluster analysis or an expert-based approach. These sharp classes are then transformed into fuzzy, multidimensional sets using the Aizerman potential function:

(4) $$\mu \left(x\right)=\left\{\begin{array}{cc} \frac{a}{d_{l}\left(1+\left(\frac{1}{b_{l}}-1\right)\cdot \frac{|x-x_{0}|}{c_{l}}\right)} & \mathrm{for}\;x<x_{0},\\ \frac{a}{d_{r}\left(1+\left(\frac{1}{b_{r}}-1\right)\cdot \frac{|x-x_{0}|}{c_{r}}\right)} & \mathrm{for}\;x\geq x_{0}, \end{array}\right.$$

where $x_{0}$ is the point of maximum membership (i.e., the representative value), and *b*, *c*, and *d* define the shape, width, and slope of the membership function on each side. The fuzziness is determined by $b_{l/r}\in \left[0,1\right]$, and the scope of uncertainty is described by $c_{l/r}>0$. The objects are unified into one-dimensional sets and then transformed into multidimensional fuzzy pattern classes using an N-fold compensatory Hamacher operator [27]:

(5) $$\mu_{\cap N}^{\mathrm{Ham}}=\frac{1}{N}\sum_{i=1}^{N}\mu_{i}.$$

During the working phase, the resulting classifier maps new, unknown samples to all defined classes and assigns them membership values. Final classification is based on the class with the highest membership. The general structure and functionality of the fuzzy pattern classification system for assessing water quality parameters have already been tested and validated in previous studies [7,28]. These publications demonstrated the model’s capability to handle uncertainty and imprecision in environmental data, confirming its suitability for real-world water monitoring applications.

To train the classifier to assess algae and harmful substances in water bodies while taking uncertainties into account, spectra were recorded for various representative substances, including spinach (as an algae analogue), phycocyanin (a blue-algae pigment), turbidity standards, uranine (a foreign substance) and pure water. The spectroscopic data is collected under controlled laboratory conditions (temperature = 22 °C, pressure = 1.013 bar). All spectra were recorded using four measurement methods: UV/Vis absorbance, fluorescence with excitation at 440 nm, fluorescence with excitation at 590 nm, and scattered light with excitation at 850 nm.

The dataset includes the following measurements:

- 40 spectra from spinach dilution series ranging from 0.25% to 40%,
- 22 spectra of phycocyanin solutions with concentrations between 10 mg L^−1^ and 700 mg L^−1^,
- 12 spectra of samples with moderate turbidity (2–10 FNU),
- 12 spectra of samples with high turbidity (10–20 FNU),
- 11 spectra of pure water,
- 22 spectra of uranine solutions at various concentrations, recorded as a foreign substance.

**Figure 4:**
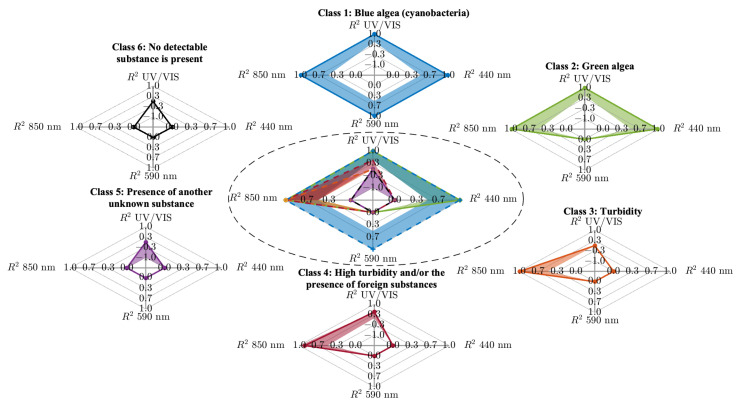
Representation of the different possible classes for detecting algae or harmful substances in water, depending on the four characteristics $R^{2}$ at UV/Vis, $R^{2}$ at LED 440 nm, $R^{2}$ at LED 590 nm and $R^{2}$ at LED 850 nm.

These spectra were preprocessed and the features extracted, as previously described. The resulting training dataset comprises 400 of these features. The extracted $R^{2}$ characteristics were then categorised into distinct groups based on the expertise of specialists in water quality assessment and spectral analysis. This expert-driven classification initially resulted in six sharply defined categories: the presence of blue algae, the presence of green algae, moderate turbidity, high turbidity and/or the presence of foreign substances (such as intensity residuals in absorbance), the presence of another unknown substance, and cases in which no detectable substance could be identified. The result is shown in Figure 4.

To model the fuzzy pattern classes, the Aizerman membership function was applied using the parameter set a=1, $b_{l/r}=0.5$, and $d_{l/r}=2$ for each object individually. These parameter values are widely recommended in the literature for the construction of normalized multivariate fuzzy membership functions, as they have proven effective in practical applications [7,29]. The corresponding class-specific fuzzy membership functions are then automatically derived based on the distribution of objects within each class. For $c_{l/r}$ an analysis of the measurement uncertainties during data acquisition and evaluation was carried out in accordance with the *Guide to the Expression of Uncertainty in Measurement* (GUM) [30,31]. The expanded uncertainty *U*, calculated following GUM guidelines, was used as the basis for setting the parameter $c_{l/r}$ in the membership function. Specifically, the expanded uncertainty was determined to be $U_{R^{2}}=5.90\%$ for UV/Vis measurements and $U_{R^{2}}=7.11\%$ for fluorescence and scattered light measurements. The detailed calculation procedure using the example of absorption is documented in [32].

**Figure 5:**
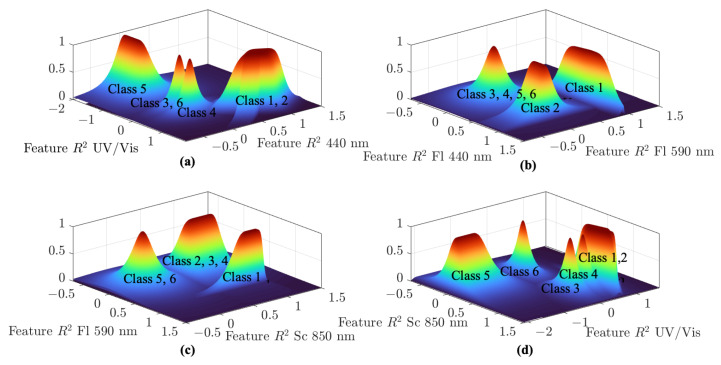
Representation of the resulting five-dimensional fuzzy pattern classifier, depicted as three-dimensional projections in subplots (**a**–**d**), where each subplot illustrates the relationship between two of the *R*^2^ features: subplot (**a**) shows *R*^2^ UV/Vis versus *R*^2^ 440 nm, subplot (**b**) shows *R*^2^ 440 nm versus *R*^2^ 590 nm, subplot (**c**) shows *R*^2^ 590 nm versus *R*^2^ Sc 850 nm, and subplot (**d**) shows *R*^2^ UV/Vis versus *R*^2^ Sc 850 nm.

The result is a five-dimensional fuzzy pattern classifier. The corresponding calculated parameters are stored in the Appendix A in Table A1. Since a complete visualization of the fuzzy classifier in five dimensions is not feasible, a breakdown into four 3D projections is presented in Figure 5, allowing for clearer interpretation and evaluation.

## 3. Results

To validate the proposed assessment method, a water sample was taken from Auensee Leipzig (Leipzig, Germany), a lake prone to cyanobacterial blooms during the summer months. These blooms have led to repeated usage restrictions over the years, posing ecological and health risks to humans and animals. Given this well-documented issue, lake Auensee is an ideal location in which to evaluate the classifier’s ability to detect harmful algae [33].

In August 2024, water samples were collected and analyzed in the laboratory. The measurement setup included UV/Vis spectroscopy, fluorescence measurements at excitation wavelengths of 440 nm and 590 nm, and scattered light measurements at 850 nm. Although the measurements were taken in a laboratory, the experimental setup closely resembles the intended in situ measurements that will be taken using the submersible probe. This enables the classifier to be tested and performed under controlled conditions before deployment in the field. It is worth noting that the sample was collected near the bank of the sea and contained a high number of particulate components, resulting in considerable turbidity. This high turbidity affected the measurements, even after applying the turbidity compensation algorithm. In extreme cases, such turbidity can mask spectral peaks entirely, making the detection of characteristic features difficult or impossible.

**Figure 6:**
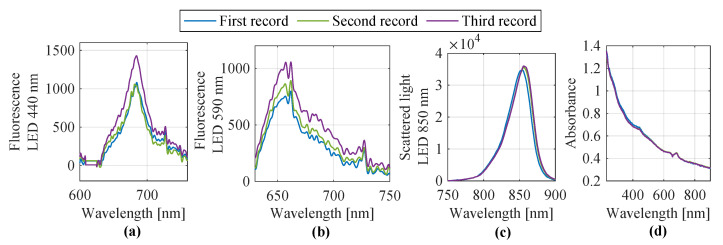
Recorded and preprocessed spectra from Lake Auensee Leipzig (August 2024) (**a**) Fluorescence spectra at an excitation wavelength of 440 nm, (**b**) fluorescence spectra at an excitation wavelength of 590 nm, (**c**) scattered light spectra at an excitation wavelength of 850 nm, and (**d**) absorbance spectra.

The acquired spectra were fully preprocessed by the described steps. The results are shown in Figure 6. Although multiple spectra were recorded, they exhibited highly similar features across all measurements. Therefore, only the first three representative spectra are presented and discussed here. The remaining spectra showed nearly identical profiles and did not provide additional information. Notably, the turbidity level displayed in Figure 6c reaches the upper limit of the measurement system (approximately 40,000 counts), which highlights the intensity of scattered light present in the sample.

Subsequently, the spectra were analyzed using the feature extraction method specific to each measurement technique. As previously discussed, despite the preliminary turbidity compensation, the UV/Vis spectra showed only low detection performance for algae-like substances. This is reflected in the low coefficients of determination ($R^{2}$ values between 0.3 and 0.4). In contrast, the fluorescence and scattered light spectra enabled clear identification of the corresponding reference spectra, with $R^{2}$ values ranging from 0.7 to 0.9. The resulting dataset (see Table 2) comprises three objects and twelve features, which can be input into the five-dimensional fuzzy pattern classifier introduced previously.

**Figure 7:**
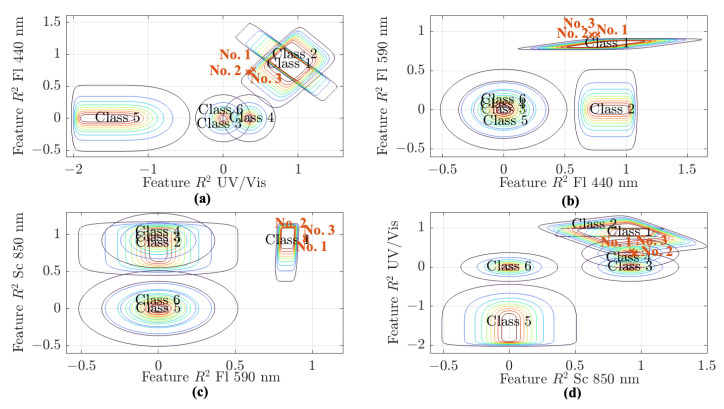
Representation of the five-dimensional fuzzy pattern classifier as three-dimensional top-view projections, showing the assigned objects from the working dataset of water samples from Lake Auensee, with each object marked by a red cross. Each subplot illustrates the relationship between two of the $R^{2}$ features: subplot (**a**) shows $R^{2}$ UV/Vis versus $R^{2}$ 440 nm, subplot (**b**) shows $R^{2}$ 440 nm versus $R^{2}$ 590 nm, subplot (**c**) shows $R^{2}$ 590 nm versus $R^{2}$ Sc 850 nm, and subplot (**d**) shows $R^{2}$ UV/Vis versus $R^{2}$ Sc 850 nm. The classes correspond to those presented in Figure 5, with the same color coding applied.

Object identification is performed again with the corresponding inherent fuzziness, derived from the calculated extended uncertainty. The classification results are shown in Figure 7 as split three-dimensional top-view representations. The precise membership values are listed in Table 3.

All three objects are assigned to Class 1 (presence of blue algae), with membership values exceeding 0.5. Compared to the values for the other classes, this clearly indicates contamination by blue algae. Therefore, the performance of the fuzzy pattern classifier can be considered validated. The consistent assignment of all objects to Class 1 confirms the fundamental validity of the classification model. As Lake Auensee is officially recognized as being contaminated with blue algae in the summer, this correct classification demonstrates that the model can reliably detect such pollution. This highlights the system’s potential to provide targeted warnings and actionable recommendations, enabling early intervention and minimizing risk.

**Figure 8:**
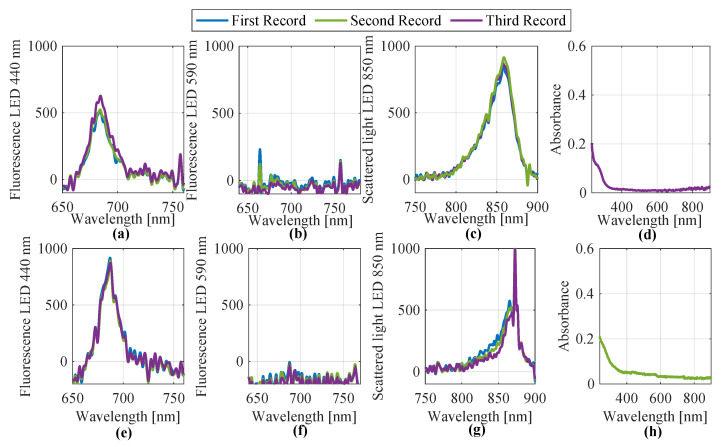
Recorded and preprocessed spectra from Lake Auensee Leipzig (September 2024) using the laboratory setup (**a**–**d**) and the submersible probe (**e**–**h**). Shown are (**a**,**e**) fluorescence spectra at an excitation wavelength of 440 nm, (**b**,**f**) fluorescence spectra at 590 nm, (**c**,**g**) scattered light spectra at 850 nm, and (**d**,**h**) absorbance spectra.

In September 2024, water samples from Lake Auensee in Leipzig were analyzed using both the laboratory-based measurement setup and a submersible probe. For the laboratory setup, UV/Vis spectroscopy and fluorescence measurements were carried out at excitation wavelengths of 440 nm, 590 nm, and 850 nm. Each sample was measured in triplicate to ensure reproducibility. Additionally, on 24 September 2024, the same lake was sampled again using a submersible probe, applying identical spectral techniques. The resulting spectra from both measurement approaches were preprocessed and are shown in Figure 8. As in the previous case, several spectra were acquired. However, due to their high similarity, only the first three are shown and used as representative examples. The top row of Figure 8 displays the four spectral groups (440 nm, 590 nm, 850 nm fluorescence, and UV/Vis absorbance) obtained from the laboratory setup, while the bottom row contains the corresponding spectra captured by the submersible probe.

Subsequently, identical feature extraction methods were applied to all eight spectra. The results for feature $R_{590}^{2}$ are zero. This is because the feature extraction algorithm is programmed to automatically assign a value of zero in cases of poor fit. The resulting $R^{2}$-based features from both measurement types are consolidated in Table 4. This dataset includes three objects (samples) for each method and a total of 36 extracted features.

These features, incorporating their respective elemental uncertainties, were evaluated using the fuzzy pattern classifier. The resulting membership values (see Table 5) show that, for both laboratory and in situ probe data, all three samples from Lake Auensee were consistently assigned to Class 2 (green algae contamination). Membership values for Class 1 (blue-green algae presence) were also notably high in both cases, indicating potential co-occurrence of both algal types.

Given these findings, a clear algal contamination can be assumed. Although the classifier assigns the dominant presence to green algae, the elevated values for blue-green algae warrant a warning and further investigation to verify the exact nature and extent of the contamination.

## 4. Discussion

### 4.1. Comparison of Data Acquisition and Evaluation Between Laboratory Test Setup and Mobile Submersible Probe

The analysis of UV/Vis and fluorescence spectra, carried out using both a laboratory setup and a mobile submersible probe, showed overall strong agreement between the recorded spectra. However, some characteristic differences were observed. These differences can be attributed to the specific features of each system and the fact that not the same water samples were measured. The mobile probe showed higher sensitivity when excited with a 440 nm LED compared to the lab setup but lower sensitivity with an 850 nm LED. These deviations are explained by differing optical properties and calibration methods of the two systems. Nevertheless, the spectra are generally comparable, highlighting the potential of the probe for real-world water sample analysis. In general, the features extracted from the probe’s spectra were lower than those from the lab setup, suggesting slight differences in light output or calibration. Slight negative shifts were also observed in the probe measurements for the 440 nm and 590 nm LEDs, but these were corrected algorithmically to ensure reliable data evaluation.

Analysis using the water assessment method at Lake Auensee produced nearly identical results for both systems, suggesting that the mobile probe can provide a similarly reliable assessment under certain conditions. Overall, the results confirm the mobile submersible probe’s suitability for on-site water analysis and its ability to accurately assess water quality parameters.

### 4.2. Discussion of the Results

A data science analysis was conducted on a water sample from Leipzig’s Auensee lake on 29 August 2024, and the system clearly identified a blue-green algae (cyanobacteria) contamination. This result aligned with an official algae warning already issued by the city of Leipzig, demonstrating the high accuracy and reliability of the classification system.

Later, on 24 September 2024, another measurement campaign was carried out. Water samples from the same lake were analyzed using both the lab setup and the submersible probe. Interestingly, both systems identified the presence of green algae this time, although high levels of blue-green algae were still detected. This change in classification may be due to recent weather conditions, such as rain and a significant drop in temperature, which could have caused the blue-green algae to die off. Additionally, a massive fish die-off was observed in the lake on 12 September 2024 [34]. A possible explanation is that dying blue-green algae consume oxygen, leading to oxygen depletion in the water, which in turn causes fish deaths. Since the measurement campaign took place after this event, the reduced blue-green algal detection and increased green algal classification could reflect the changed environmental conditions. Despite these shifts, the fuzzy pattern classifier proved to be highly reliable, adapting well to different environmental scenarios. Under warmer, stable summer conditions, it consistently identified blue-green algae, while under cooler, post-rainfall conditions, it adjusted its classification accordingly.

Compared to other optical machine learning systems, such as portable Raman/fluorescence platforms or recent machine learning-based bloom monitoring frameworks [35,36], our system offers a distinct approach by combining UV/Vis and fluorescence spectroscopy with sequential light-emitting diode excitation. This setup enables pigment-specific signals to be isolated without overlapping peaks. Sequential acquisition, together with full-spectrum feature extraction, enhances the reliability of classification even under co-occurring blooms or when pigments are degraded. Additionally, the fuzzy pattern classifier explicitly accounts for measurement uncertainty, enabling the system to adapt to variable environmental conditions. Although stochastic generalisation models in Raman spectroscopy provide robust detection in complex matrices, our methodology is specifically designed for in situ aquatic monitoring and has been shown to perform well in real-world field campaigns. Coupled with a mobile submersible probe and careful data pre-processing, the system can detect both cyanobacterial and green algal blooms sensitively and adaptively. Overall, these features establish a scalable, field-deployable platform that combines spectral modality, uncertainty handling and practical environmental application. This complements existing monitoring strategies and provides a valuable tool for early warning and water quality management.

## 5. Conclusions

This study presents an approach for in situ water quality monitoring, focusing on the detection and classification of algal and harmful substances in water bodies, with explicit consideration of measurement uncertainties. Although the study focused specifically on blue-green (cyanobacteria) and green algae, this approach can also be used to detect other substances, such as turbidity and harmful or unknown substances. The system combines UV/Vis and fluorescence spectroscopy with a fuzzy pattern classifier to enable reliable operation in laboratory and real-world environments. Comprehensive pre-processing steps are applied to the data, including spectral normalization, noise reduction, and correction of systematic deviations, to minimize measurement uncertainty and enhance signal quality. These measures ensure that the input data is well prepared for accurate analysis. Furthermore, a feature extraction strategy that evaluates the full spectral profile is used, rather than focusing on individual wavelengths or peak intensities. This allows subtle yet meaningful spectral patterns associated with different types of algae to be identified. To evaluate the system’s transferability to field applications, measurements were first conducted using a laboratory setup and subsequently with a developed submersible probe designed for in situ deployment. Although minor deviations were observed between the two systems due to environmental and optical differences, the classification results remained consistent. Explicitly addressing measurement uncertainties throughout the data processing and classification stages enables the system to achieve greater robustness and reliability when interpreting complex spectral data. The results from laboratory and in situ measurement systems confirm that the classifier can adapt to environmental variability and deliver accurate, consistent results, even under challenging conditions.

Overall, combining a mobile submersible probe with refined spectral pre-processing, comprehensive feature analysis, and uncertainty-aware classification creates a robust basis for modern water quality monitoring. This system is particularly valuable for environmental surveillance and the early detection of harmful algae, providing a practical tool for the sustainable management of aquatic ecosystems in the face of growing ecological and climate-related challenges.

While this study demonstrates the potential of the proposed system, further research is needed to validate its performance in different bodies of water and environmental conditions. In particular, more in situ measurements from varied, ecologically diverse sites are essential to improve the robustness and generalizability of the classification.

Future work should also include long-term field studies, particularly during different bloom stages, as well as expanding the training dataset to enhance classification accuracy. Additionally, integrating advanced algorithms and exploring additional spectral features could enhance sensitivity and reliability further. Continued development and field validation are therefore crucial steps towards establishing a scalable, practical tool for the early detection of harmful algal blooms and improved water quality monitoring.

## Figures and Tables

**Table 1:** Overview of various studies addressing uncertainty considerations in UV/Vis and fluorescence spectroscopy.

Technique	Focus on Uncertainty	Ref.
UV/Vis	An overview of the eight key sources of uncertainty and their evaluation is provided. For example, the influence of factors such as the repeatability of spectrophotometer readings, spectrophotometer drift, stray light, interference from the constituents of the matrix and the decomposition of the photometric complex is discussed. However, these factors are not integrated into the assessment method.	[9]
UV/Vis	The uncertainty due to hydrological and site-specific factors in phosphorus prediction using UV-Vis spectroscopy is described.	[10]
UV/Vis	The utilization of local calibration has been identified as a method for the purpose of reducing measurement uncertainty in the aftermath of storm events.	[11]
UV/Vis	A comparison of calibration methods was conducted, and it was determined that Partial Least Squares is optimal for practical use as a linear regression method.	[12]
UV/Vis	Improved accuracy in wastewater analysis through specific calibration and consideration of spectral artefacts and laboratory-related errors.	[13]
Fluorescence	A correction factor is used to compensate for interference from chlorophyll, but its effectiveness is limited under field conditions.	[14]
Fluorescence	Models and sampling designs, as well as instrument adjustments, were used to reduce fluorescence quenching due to light exposure.	[15]
Fluorescence	Recommendation for laboratory-based compensation for temperature and turbidity interference and for concurrent turbidity/temperature measurement.	[16]
Fluorescence	Use uncertainty propagation to estimate concentration uncertainty. Reagent-specific calibration is advised.	[17]
Fluorescence	A multi-parameter probe is required for BOD_5_ estimation in treated wastewater, as local calibration is needed due to increased uncertainty.	[18]
Fluorescence	A comparison with other sensors reveals that there are uncertainties due to unfiltered samples and that further investigation is needed.	[19]
Fluorescence	The optical absorption and fluorescence properties of dissolved organic matter are influenced by various factors. Fluorescence measurements have been shown to correlate directly with absorption coefficients, thereby reducing uncertainties in quantum yield determination.	[20]

**Table 2:** Calculated $R^{2}$ features from fluorescence spectra (440 nm, 590 nm, 850 nm) and UV/Vis absorbance for each object.

Object No.	$R^{2}$ 440 nm	$R^{2}$ 590 nm	$R^{2}$ 850 nm	$R^{2}$ UV/Vis
1	0.7679	0.9627	0.9287	0.4017
2	0.7202	0.9605	0.9512	0.3394
3	0.7206	0.9571	0.9490	0.3605

**Table 3:** Calculated membership values μ of objects from the Auensee measurement (29 August 2024) assigned to different classes.

Object No.	Assigned Class	$\mu_{1}$	$\mu_{2}$	$\mu_{3}$	$\mu_{4}$	$\mu_{5}$	$\mu_{6}$
1	Class 1	0.701	0.077	0.033	0.037	0.018	0.022
2	Class 1	0.555	0.077	0.036	0.039	0.019	0.023
3	Class 1	0.590	0.077	0.036	0.039	0.019	0.023

$\mu_{i}$ denotes the degree of membership to Class *i*. The classification was performed using the described fuzzy pattern classifier in Figure 5.

**Table 4:** Resulting $R^{2}$ values for features derived from UV/Vis and fluorescence spectra obtained from Lake Auensee samples using both the laboratory setup and the submersible probe.

Object No.	$R^{2}$ 440 nm	$R^{2}$ 590 nm	$R^{2}$ 850 nm	$R^{2}$ UV/Vis
Laboratory Setup				
1	0.8365	0	0.9553	0.6750
2	0.8291	0	0.9667	0.6646
3	0.8654	0	0.9632	0.6534
Submersible Probe				
1	0.8894	0	0.6294	0.4553
2	0.8765	0	0.5236	0.5337
3	0.8840	0	0.4003	0.5861

**Table 5:** Calculated membership values μ of all objects from Lake Auensee measured using both the laboratory setup and the submersible probe.

Object No.	Class Assignment	$\mu_{1}$	$\mu_{2}$	$\mu_{3}$	$\mu_{4}$	$\mu_{5}$	$\mu_{6}$
Laboratory Setup							
1	Class 2	0.892	0.984	0.045	0.066	0.017	0.026
2	Class 2	0.879	0.978	0.046	0.068	0.018	0.027
3	Class 2	0.883	0.980	0.045	0.063	0.017	0.026
Submersible Probe							
1	Class 2	0.427	0.604	0.050	0.062	0.023	0.039
2	Class 2	0.357	0.610	0.045	0.057	0.023	0.041
3	Class 2	0.255	0.453	0.039	0.050	0.022	0.042

$\mu_{i}$ denotes the degree of membership to Class *i*. The classification was performed using the described fuzzy pattern classifier in Figure 5.

## Data Availability

The analytical results and data are available from the corresponding author upon request.

## Abbreviations

The following abbreviations are used in this manuscript:

UV/Vis	Ultraviolet/Visible
GUM	Guide to the expression of uncertainty in measurement

## Appendix A

**Table A1:** Calculated parameters for the construction of the five-dimensional fuzzy pattern classifier used for detecting harmful algae and other substances.

Class	Feature No.	$x_{0}$	Angle	$b_{l}$	$b_{r}$	$c_{l}$	$c_{r}$	$d_{l}$	$d_{r}$
Class 1	1	0.954	1.000	0.367	0.398	0.365	0.225	8.670	8.100
Class 1	2	0.974	0.243	0.336	0.043	0.195	0.098	6.584	17.634
Class 1	3	0.841	1.237	0.090	0.359	0.112	0.181	20.000	9.849
Class 1	4	0.960	0.270	0.469	0.316	0.209	0.159	4.201	10.305
Class 2	1	0.951	1.000	0.105	0.309	0.128	0.260	16.678	5.830
Class 2	2	0.976	−0.077	0.152	0.031	0.123	0.096	12.926	18.787
Class 2	3	0.000	0.000	0.496	0.496	0.071	0.071	2.000	2.000
Class 2	4	0.952	−1.381	0.422	0.074	0.185	0.098	7.163	20.000
Class 3	1	0.000	1.000	0.500	0.500	0.050	0.050	2.000	2.000
Class 3	2	0.000	0.000	0.500	0.500	0.050	0.050	2.000	2.000
Class 3	3	0.000	0.000	0.500	0.500	0.050	0.050	2.000	2.000
Class 3	4	0.964	0.000	0.500	0.500	0.050	0.050	2.000	2.000
Class 4	1	−1.297	1.000	0.500	0.500	0.050	0.050	2.000	2.000
Class 4	2	0.000	0.000	0.500	0.500	0.050	0.050	2.000	2.000
Class 4	3	0.000	0.000	0.500	0.500	0.050	0.050	2.000	2.000
Class 4	4	0.000	0.000	0.500	0.500	0.050	0.050	2.000	2.000
Class 5	1	0.000	1.000	0.500	0.500	0.050	0.050	2.000	2.000
Class 5	2	0.000	0.000	0.500	0.500	0.050	0.050	2.000	2.000
Class 5	3	0.000	0.000	0.500	0.500	0.050	0.050	2.000	2.000
Class 5	4	0.000	0.000	0.500	0.500	0.050	0.050	2.000	2.000

Parameters shown: representative value $x_{0}$, angle, slope parameters $b_{l}$/$b_{r}$, core widths $c_{l}$/$c_{r}$, and support widths $d_{l}$/$d_{r}$ used for fuzzy membership modeling.
